# Whole-genome sequencing of *Bifidobacterium breve* Y490 and *Bifidobacterium longum* subsp. *infantis* E16 isolated from infant feces

**DOI:** 10.1128/mra.00595-26

**Published:** 2026-07-22

**Authors:** Irene Boscá-Sánchez, Eva M. Moya-Gonzálvez, Jesús Rodríguez-Díaz, María J. Yebra

**Affiliations:** 1 Departamento de Biotecnología de Alimentos, IATA-CSIC, Laboratorio de Bacterias Lácticas y Probióticos, Valencia, Spain; 2 Departamento de Microbiología, Facultad de Medicina, Universidad de Valencia 16781, Valencia, Spain; 3 INCLIVA. Instituto de Investigación Sanitaria del Hospital Clínico de Valencia, Valencia, Spain; Nanchang University, Nanchang, Jiangxi, China

**Keywords:** genome, *Bifidobacterium breve*, *Bibifobacterium infantis*, whole sequence

## Abstract

We report the complete genome sequences of *Bifidobacterium breve* Y490 and *Bifidobacterium longum* subsp. *infantis* E16. These genomic resources will facilitate research into the mechanisms underlying the utilization of glycoconjugate-derived substrates and the potential application of these strains in synbiotic formulations.

## ANNOUNCEMENT

*Bifidobacterium breve* and *Bifidobacterium longum* subsp. *infantis* are well-recognized probiotics with notable benefits for human health (1, 2). Milk-derived *N*-glycans can support bifidobacterial growth (3). Here, we identified two *Bifidobacterium* strains that grow efficiently in a bovine milk protein isolate treated with glycosidases to release *N*-glycans from glycoproteins.

*Bifidobacterium breve* Y490 was isolated from a disaccharide fermentation culture inoculated with fecal microbiota from breast-fed infants (4). *Bifidobacterium infantis* E16 was isolated on Man, Rogosa, and Sharp medium (MRS) agar under anaerobic conditions (37°C, 48 h) from a pooled fecal sample of four infants. Samples were preserved at −80°C in brain heart infusion, 20% skimmed milk, and 0.1% cysteine before isolation. Taxonomic identification of *B. breve* Y490 was performed as previously described (4), and the 16S rRNA gene sequence was deposited in GenBank (MN650613). *B. infantis* E16 was identified by 16S rRNA gene amplification using primers 27F (5) and 924R (6). BLAST analysis against the National Center for Biotechnology Information (NCBI) database confirmed its identity, with the closest match being *B. infantis* NCTC13219 (UAQL01000033.1), showing 99.9% nucleotide identity over 100% query coverage. The sequence was deposited under accession number OR608294. Genomic DNA was extracted from 24 h cultures grown in MRS at 37°C using the MasterPure DNA Extraction Kit (Epicenter), including lysozyme and mutanolysin pretreatment followed by glass-bead disruption.

The genomes of *B. breve* Y490 and *B. infantis* E16 were sequenced using the PacBio VegaSystem at the Genomics Unit of the SCSIE of the University of Valencia (Spain). Sequencing libraries were prepared using the SMRTbell Prep Kit 3.0 (Pacific Biosciences). Genomic DNA was sheared to 7–10 kb, repaired, adapter-ligated, size-selected with AMPure PB beads, and sequenced on the PacBio VEGA System using VEGA polymerase-bound SMRTbell templates loaded onto a Vega SMRT Cell. Sequencing was conducted in HiFi (Circular Consensus Sequencing) mode with a 24-h movie time, utilizing real-time base-calling to generate high-accuracy (>Q20) long reads. Raw read quality control was performed within the SMRT Link v25.3 during HiFi (CCS) read generation. Only reads meeting the Q20 threshold were retained for downstream analyses, and sequencing quality was assessed using PacBio metrics, including read yield, read length distribution, N50, and quality scores. The microbial analysis pipeline (7) was employed for *de novo* assembly and DNA methylation detection. The workflow uses the Improved Phased Assembler (IPA2, SL-release-10.2.1-19-g9f2dfc), and assemblies were automatically circularized by trimming terminal redundancies and subsequently rotated to the predicted origin of replication (oriC). The circular chromosome of *B. breve* Y490 was 2,299,569 bp long, and that of *B. infantis* E16 was 2,636,434 bp long (Table 1). The complete genome sequences were functionally annotated using Bakta v1.11.4 (8). The analysis was supported by the Bakta full database v6.0, providing comprehensive identification of protein-coding sequences (CDS), tRNA, ribosomal RNAs (rRNA), transfer-messenger RNAs (tmRNA), and other non-coding features. Predicted protein-coding genes were cross-referenced against UniProt, RefSeq and COG for functional characterization. A total of 1,928 protein-coding genes were identified for *B. breve* Y490 and 2,276 for *B. infantis* E16 (Table 1). Default parameters were used except where otherwise noted.

**TABLE 1:** Genome features of *B. breve* Y490 and *B. infantis* E16^*a*^

Feature	*B. breve* Y490	*B. infantis* E16
HiFi reads	4.0 M	3.97 M
HiFi read yield	23.26 Gb	23.39 Gb
HiFi read length (mean)	5.80 kb	5.88 kb
HiFi read length N50	6.30 kb	6.41 kb
Coverage	10,114×	8,869×
Contigs	1	1
Genome length	2,299,569 bp	2,636,434 bp
GC content	58.7%	59.7%
CDS	1,928	2,276
tRNA genes	53	57
rRNA genes	6	12

^*a*^ CDS, coding DNA sequence.

## Data Availability

The genome sequences and annotations of *Bifidobacterium breve* Y490 and *Bifidobacterium longum* subsp. *infantis* E16 have been deposited in DDBJ/ENA/GenBank under accession numbers JBYEPR000000000 and JBYEPS000000000, respectively. Raw sequence reads are deposited at the NCBI Sequence Read Archive under accession numbers SRR37421585 and SRR37422265, respectively. The associated BioProject accession numbers are PRJNA1430324 and PRJNA1430382, respectively.
